# Equine Corneal Wound Healing Using Mesenchymal Stem Cell Secretome: Case Report

**DOI:** 10.3390/ani14131842

**Published:** 2024-06-21

**Authors:** Alejandro Casado-Santos, Elsa González-Cubero, Maria Luisa González-Fernández, Yaiza González-Rodríguez, Mª Belén García-Rodríguez, Vega Villar-Suárez

**Affiliations:** 1 Department of Surgery, Medicine and Veterinary Anatomy, Faculty of Veterinary Sciences, Campus de Vegazana, University of Léon, 24071 Léon, Spain; acasas@unileon.es (A.C.-S.); mlgon@dentalebiomedica.com (M.L.G.-F.); ygonzr03@estudiantes.unileon.es (Y.G.-R.); mbgarr@unileon.es (M.B.G.-R.); 2 Department of Neurosurgery, Stanford School of Medicine, Stanford University, Palo Alto, CA 94305, USA; elsagonz@stanford.edu; 3 Institute of Biomedicine (IBIOMED), Faculty of Veterinary Sciences, Campus de Vegazana, University of León, 24071 León, Spain

**Keywords:** corneal non-healing ulcer, horses, adipose tissue mesenchymal stem cells, secretome

## Abstract

**Simple Summary:**

This case report describes the successful treatment of a non-healing corneal ulcer in a 28-year-old mare using the secretome derived from adipose tissue-derived mesenchymal stem cells (ASCs). Despite initial conventional treatment with antibiotics, anti-inflammatory drugs, and surgical debridement, the corneal ulcer failed to heal properly. As an alternative regenerative approach, the ASC secretome, containing trophic factors, cytokines, and extracellular vesicles, was topically administered to the affected eye. Remarkably, within one week of secretome treatment, the clinical signs resolved, and the corneal ulcer exhibited complete re-epithelialization, regained transparency, and reduced neovascularization. No recurrence was observed during the 1.5-year follow-up period. This highlights the potential of the ASC secretome as a novel cell-free therapy for treating refractory corneal ulcers in horses.

**Abstract:**

Corneal ulcers are a common and potentially vision-threatening condition in horses that can be challenging to treat with conventional therapies alone. This case report describes the successful treatment of a non-healing corneal ulcer in a 28-year-old Hispano-Bretón mare using the secretome derived from adipose tissue-derived mesenchymal stem cells (ASCs). Despite initial treatment with antibiotics, anti-inflammatory drugs, and surgical debridement, the corneal ulcer failed to heal properly, exhibiting persistent epithelial defects and stromal complications. As an alternative regenerative approach, the ASC secretome, a rich source of trophic factors, cytokines, and extracellular vesicles, was topically administered to the affected eye. Remarkably, within one week of secretome treatment, the clinical signs of blepharospasm and epiphora resolved, and the corneal ulcer exhibited complete re-epithelialization, regained transparency, and reduced neovascularization. No recurrence was observed during the 1.5-year follow-up period. This case highlights the potential therapeutic benefits of the ASC secretome in promoting corneal wound healing and suggests its promise as a novel cell-free therapy for treating refractory corneal ulcers in horses.

## 1. Introduction

Corneal ulcer is a common and potentially serious condition in horses. Damage to the cornea, resulting in the loss of epithelial cells with exposed corneal stroma, creates an ulcer on the corneal surface [1]. Squinting (blepharospasm) or keeping an eye shut and tearing (epiphora) are often the first signs that owners will notice in pets with corneal ulcers. Fluorescein stain uptake in the ulcerated region confirms the diagnosis of an ulcer. The categorization of the ulcer is based on a combination of clinical signs and the visual inspection of the eye [1,2].

Indolent corneal ulcers, also known as refractory ulcers, “boxer” ulcers, spontaneous chronic corneal epithelial defects (SCCEDs), and recurrent erosions, are a special type of superficial ulcer that fails to resolve through the normal wound-healing process [3]. Hallmark clinical and histologic features of SCCEDs include a superficial corneal ulcer that (1) does not extend into the stroma; (2) is associated with redundant, non-adherent corneal epithelial borders that may be associated with an acellular hyaline zone in the anterior stroma; (3) persists for weeks or months if not adequately addressed; and (4) may or may not include neovascularization and edema [4,5,6].

The underlying pathophysiology of SCCEDs is not completely understood, and there is some thought that these ulcers may be the shared end result of a variety of pathways. Ultimately, indolent corneal ulcers result from the dysmaturation of corneal epithelia that do not properly attach to the underlying stroma of the eye, creating a lesion composed of loose, poorly adhered epithelia overlying corneal stroma (lipping) [5,7].

Traditional treatments for corneal ulcer in horses include antibiotics, anti-inflammatory medications, and surgery [1,7]. However, these treatments can be costly, time-consuming, and sometimes ineffective [8]. In recent years, regenerative medicine has emerged as a promising approach for treating various conditions, including corneal wounds [9]. One of the most promising alternatives developed is the use of mesenchymal stem cells (MSCs) [10,11,12]. In MSCs therapy, stem cells are harvested from the patient or from a donor and then cultured in a laboratory. The MSCs are then administered to the patient to promote healing and tissue regeneration. However, recent studies have shown that the beneficial effects of MSCs therapy may not be solely due to the stem cells themselves but rather to the secretion of various factors, known as the secretome, also called conditioned medium (CM), by the MSCs [13,14]. MSCs secretome refers to the collection of bioactive molecules, including growth factors, cytokines, and extracellular vesicles (EVs), secreted by these cells [15]. The secretome of MSCs has gained significant attention due to its potential to modulate the wound healing process by regulating various cellular processes, such as inflammation, angiogenesis, and extracellular matrix remodeling [16]. Furthermore, the use of the secretome eliminates the need for direct cell transplantation, reducing the risks associated with cell-based therapies, such as immune rejection and potential tumorigenicity [17].

This case report describes a novel approach to treating a corneal wound in a Hispano-Bretón mare using the secretome derived from adipose tissue mesenchymal stem cells (ASCs). We describe the clinical presentation, treatment protocol, and outcome, highlighting the potential of the ASC secretome as a promising therapeutic strategy for corneal wound healing in veterinary ophthalmology.

## 2. Case Description

A 28-year-old mare of the Hispano-Bretón breed was kept in a paddock with two other horses. The general examination of the animal does not show any alteration, the ocular examination shows blepharospasm, moderate blepharitis, and moderate/mild epiphora, and the tear secretion is serous. The pupil appears miotic, with moderate corneal edema and localized corneal neovascularization in the dorsal region, with conjunctival hyperemia. Fluorescein staining revealed the presence of a corneal ulcer of about 7 mm in diameter, apparently uncomplicated.

Treatment was started with tobramycin in eye drops (Tobrex^®^, Novartis, Basilea, Switzerland) every 4 h, atropine (Colircusí^®^ Atropina 1%, NTC, Madrid, Spain) in eye drops every 12 h during the first 48 h and then as needed, autologous serum every 2 h in the morning and every 3 h in the afternoon, and intravenous flunixin meglumine (Finadyne^®^, MSD, Rahway, NJ, USA) at a dose of 1.1 mg/kg every 24 h for two days.

**Figure 1:**
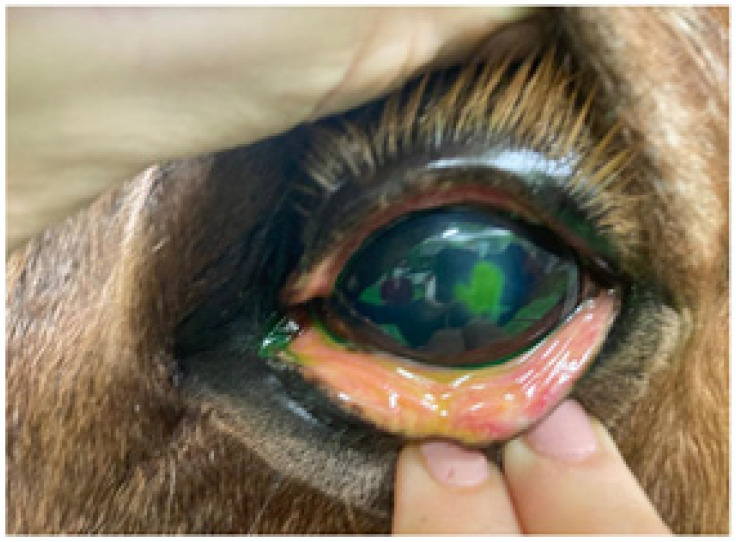
Fluorescein test stain showed corneal injury with fluorescein infiltration under the edges of the epithelium.

A first review after 4 days showed an improvement in blepharospasm and corneal edema, although epiphora persisted. Fluorescein staining revealed dye infiltration under the epithelial borders of the lesion. Slit-lamp biomicroscopic examination and fluorescein staining revealed detached epithelial edges (Figure 1), confirming the diagnosis of indolent/nonhealing corneal ulcer. The examination was completed, ruling out the existence of a foreign body, and debridement was performed. After sedation with detomidine (Domosedan^®^, Orion, Espoo, UK) and butorphanol (Torbugesic Vet^®^, Zoetis, Madrid, Spain), in addition to topical anesthesia, the mechanical debridement of the lesion was performed with a Algerbrush 3.5 mm^®^ diamond bur. The non-adherent epithelium was removed along the entire contour of the lesion, and the lesion bed was also reamed. 

**Figure 2:**
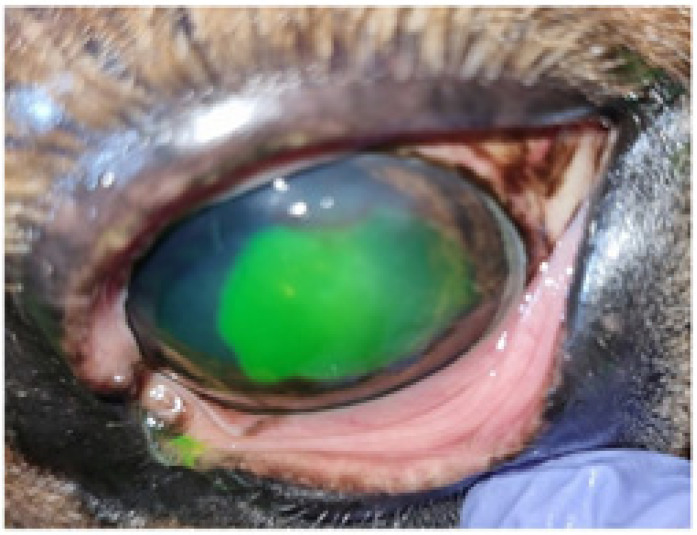
Aspect of the lesion after the first debridement, a significant increase in the extent of the corneal ulcer is observed.

The result was a significant increase in the extent of the corneal ulcer. It occupied two-thirds of the corneal surface (Figure 2). The previously prescribed medical treatment with systemic flunixin meglumine was maintained for two days.

**Figure 3:**
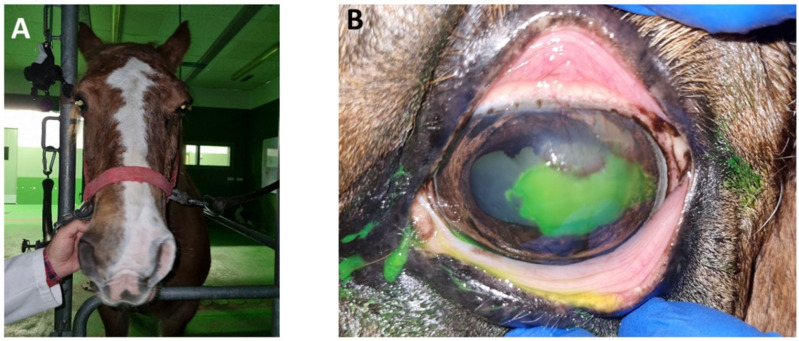
Aspect of the lesion at the second examination 1 week later. (**A**) Moderate blepharospasm in the left eye was observed. (**B**) After the second debridement, the lesion was fluorescein positive.

At the next examination 1 week later, moderate blepharospasm (Figure 3A) was observed. The lesion was fluorescein-positive (Figure 3B) and was accompanied by significant cellular and vascular infiltration in the superotemporal quadrant of the cornea. She underwent another debridement with a diamond burr. The result was a reduction in the ulcerated surface with respect to the previous debridement. Medical treatment was maintained, in this case with flunixin meglumine, for 3 days.

**Figure 4:**
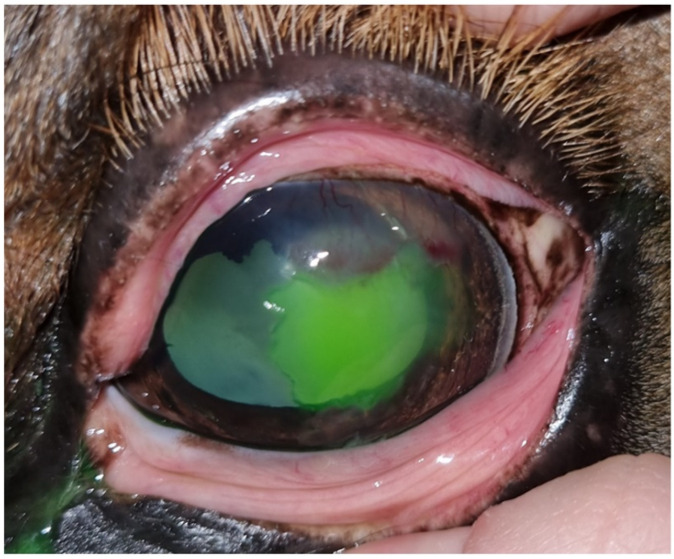
After all antibiotic and anti-inflammatory treatment and third debridement, the lesion persists without significant improvement with respect to the previous debridement.

One week later, at the second examination, the clinical signs of blepharospasm and epiphora were still present, with significant cellular and vascular infiltration of the cornea. Fluorescein staining was negative, but the epithelium did not appear to be completely adherent to the stroma on biomicroscopic examination, and a touch with the Kimura spatula revealed the sliding of the epithelium over the corneal stroma. Debrided, the final result is a lesion of greater extent than after the previous debridement (Figure 4). There is no improvement from the previous consultation.

After this conventional treatment without significant evolutions, we consider the need to modify the therapeutic strategy in order to promote proper re-epithelialization, so the patient underwent therapeutic intervention with ASC secretome. The ASCs used for generating the secretome were isolated from adipose tissue collected from healthy donor horses during routine surgical procedures at our facility. The donor horses were of various breeds, with ages ranging from 5 to 12 years old, and had no underlying health conditions. Specifically, the ASCs used in this case were derived from a 7-year-old Hispano-Bretón mare that was a healthy surgical donor. The adipose tissue was obtained from subcutaneous tissue. ASCs were isolated from the adipose tissue through enzymatic digestion with collagenase at 37 °C for 2 h and then they were cultured in standard conditions at 37 °C, 5% CO_2_, and 95% humidity. The cells were expanded until they reached 80% confluence, which typically took 10–15 days depending on the initial seeding density. When 80% confluence was attained, ASCs were maintained in serum-free DMEM medium for 48 h to produce the secretome. The CM was collected and centrifuged at 2000× *g* for 20 min to remove cellular debris. The cell-free CM, containing the secretome of proteins, cytokines, and other factors secreted by the equine ASCs, was concentrated using 3 kDa ultrafiltration membranes (Amicon Ultra-15, Millipore^®^, Merck, MA, USA) by centrifugation at 4000× *g* for 30 min. The concentrated secretome was diluted in sterile saline to a final protein concentration of 1 mg/mL, as determined by the BCA assay. The secretome was administered topically to the affected eye as 1 drop every 4 h throughout the day until complete corneal re-epithelialization was achieved. The owners were informed of the experimental treatment condition, and their consent was obtained.

**Figure 5:**
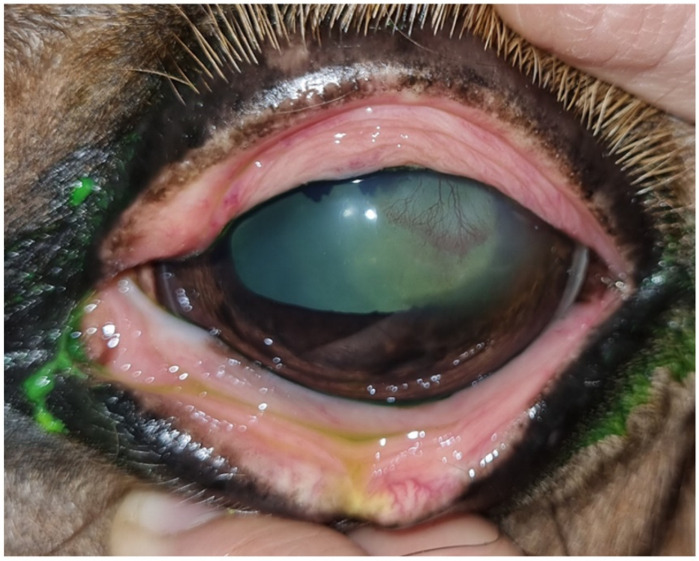
Negative fluorescein test stain showed the absence of corneal injury after secretome therapy. The cornea has regained transparency, reduced neovascularization, and disappeared cellular infiltration.

One week later, the clinical signs of blepharospasm and epiphora had disappeared and the lesion was fluorescein-negative (Figure 5). On biomicroscopic examination, the lesion appeared well-re-epithelialized, yet swabbing was performed on the surface of the lesion, which revealed the adherence of the epithelium to the corneal stroma.

The re-epithelialized area had regained transparency, cellular infiltration had disappeared, and improved corneal neovascularization was observed. The administration of secretome was maintained for 3 more days, and the patient was discharged with no recurrence in a year and a half after treatment. No adverse effects were observed following the topical administration of the allogeneic ASC secretome to the affected eye. The treatment was well-tolerated, and no signs of inflammation, discomfort, or other complications were noted during the follow-up period.

## 3. Discussion

The findings from this case report highlight the potential therapeutic benefits of using mesenchymal stem cell (MSC) secretome, specifically from adipose-derived MSCs (ASCs) [18], for treating non-healing corneal ulcers in horses [9,19]. Despite initial conventional treatment with antibiotics, anti-inflammatory drugs, and debridement, the corneal ulcer in this Hispano-Bretón mare failed to heal properly, exhibiting signs of persistent epithelial defects.

The lack of significant improvement with standard therapy prompted the exploration of an alternative regenerative approach using the secretome derived from cultured equine ASCs [3]. The ASC secretome, a rich milieu of trophic factors, cytokines, and extracellular vesicles, has been shown to modulate various cellular processes crucial for wound healing, including inflammation, angiogenesis, and extracellular matrix remodeling [20,21].

By harnessing the paracrine effects of the ASC secretome, topical administration facilitated the re-epithelialization of the corneal ulcer and the resolution of associated clinical signs within a week of treatment initiation.

These findings corroborate previous studies that have demonstrated the therapeutic potential of MSC secretomes in promoting corneal wound healing [22]. For instance, a study by Saccu et al. reported significantly faster healing times and reduced complications in corneal ulcers treated with bone marrow-derived MSC secretome compared to a placebo group [23]. Similarly, Cunha et al. [24] observed enhanced corneal re-epithelialization and reduced inflammation in rats treated with ASC secretome after corneal alkali burns. However, there is a paucity of research investigating the use of stem cell therapies for the treatment of corneal ulcers in veterinary medicine. This gap in knowledge is even more pronounced when considering the equine species. To date, no studies have evaluated the therapeutic effects of stem cell secretomes, specifically those derived from ASCs, on corneal ulcers in horses.

The regenerative capacity of MSC secretomes is attributed to their ability to modulate the wound microenvironment through various mechanisms [21]. The secreted factors can exert anti-inflammatory effects by regulating the activity of immune cells and reducing oxidative stress. Additionally, the secretome contains growth factors and cytokines that stimulate the proliferation and migration of corneal epithelial cells, facilitating re-epithelialization [25,26,27]. Furthermore, the extracellular vesicles present in the secretome can transfer bioactive molecules, such as proteins, lipids, and nucleic acids, to target cells, influencing their behavior and promoting tissue repair [28,29].

While the results from this case report are promising, it is important to acknowledge the limitations of a single case study. Further controlled studies with larger sample sizes are necessary to validate the efficacy and safety of ASC secretome therapy for treating corneal ulcers in horses. Additionally, optimizing the dosing regimen, administration route, and characterization of the secretome could potentially enhance its therapeutic effects.

## 4. Conclusions

Overall, these findings suggest that ASC secretome therapy may be a safe and effective novel regenerative therapy for treating non-healing corneal ulcers in horses. The topical application of the ASC secretome facilitated corneal re-epithelialization and the resolution of clinical signs in a case refractory to conventional treatment. These findings contribute to the growing body of evidence supporting the use of MSC secretomes as a cell-free, minimally invasive approach for promoting tissue repair and regeneration in veterinary ophthalmology.

## Data Availability

Data will be made available on request.

## References

[1] Hartley C. (2015). Differential Diagnosis and Management of Corneal Ulceration in Horses, Part 2. In Practice.

[2] Michau T.M., Schwabenton B., Davidson M.G., Gilger B.C. (2003). Superficial, Nonhealing Corneal Ulcers in Horses: 23 Cases (1989–2003). Vet. Ophthalmol..

[3] Prucha V.J.S., Tichy A., Nell B. (2020). Equine Non-Healing Corneal Ulcers: A Retrospective Evaluation of 57 Cases (2001–2017). Tierarztl. Prax. Ausg. G Grosstiere. Nutztiere..

[4] Williams L.B., Pinard C.L. (2013). Corneal Ulcers in Horses. Compend. Contin. Educ. Vet..

[5] Cooley P.L., Wyman M. (1986). Indolent-like Corneal Ulcers in 3 Horses. J. Am. Vet. Med. Assoc..

[6] Hempstead J.E., Clode A.B., Borst L.B., Gilger B.C. (2014). Histopathological Features of Equine Superficial, Nonhealing, Corneal Ulcers. Vet. Ophthalmol..

[7] Mohan R.R., Kempuraj D., D’Souza S., Ghosh A. (2022). Corneal Stromal Repair and Regeneration. Prog. Retin. Eye Res..

[8] Morgan R.V., Abrams K.L. (1994). A Comparison of Six Different Therapies for Persistent Corneal Erosions in Dogs and Cats. Vet. Comp. Ophthalmol..

[9] Falcão M.S.A., Brunel H.D.S.S., Peixer M.A.S., Dallago B.S.L., Costa F.F., Queiroz L.M., Campbell P., Malard P.F. (2020). Effect of Allogeneic Mesenchymal Stem Cells (MSCs) on Corneal Wound Healing in Dogs. J. Tradit. Complement. Med..

[10] Gugjoo M.B. (2022). Mesenchymal Stem Cells Therapeutic Applications in Eye and Adnexa Ailments. Therapeutic Applications of Mesenchymal Stem Cells in Veterinary Medicine.

[11] Singh V.K., Sharma P., Vaksh U.K.S., Chandra R. (2022). Current Approaches for the Regeneration and Reconstruction of Ocular Surface in Dry Eye. Front. Med..

[12] Alió del Barrio J.L., De la Mata A., De Miguel M.P., Arnalich-Montiel F., Nieto-Miguel T., El Zarif M., Cadenas-Martín M., López-Paniagua M., Galindo S., Calonge M. (2022). Corneal Regeneration Using Adipose-Derived Mesenchymal Stem Cells. Cells.

[13] Xia J., Minamino S., Kuwabara K., Arai S. (2019). Stem Cell Secretome as a New Booster for Regenerative Medicine. Biosci. Trends.

[14] Makridakis M., Roubelakis M.G., Vlahou A. (2013). Stem Cells: Insights into the Secretome. Biochim. Biophys. Acta.

[15] Al Naem M., Bourebaba L., Kucharczyk K., Röcken M., Marycz K. (2020). Therapeutic Mesenchymal Stromal Stem Cells: Isolation, Characterization and Role in Equine Regenerative Medicine and Metabolic Disorders. Stem Cell Rev. Rep..

[16] Keshtkar S., Azarpira N., Ghahremani M.H. (2018). Mesenchymal Stem Cell-Derived Extracellular Vesicles: Novel Frontiers in Regenerative Medicine. Stem Cell Res. Ther..

[17] Pawitan J.A. (2014). Prospect of Stem Cell Conditioned Medium in Regenerative Medicine. Biomed Res. Int..

[18] Bellei B., Migliano E., Tedesco M., Caputo S., Papaccio F., Lopez G., Picardo M. (2018). Adipose Tissue-Derived Extracellular Fraction Characterization: Biological and Clinical Considerations in Regenerative Medicine. Stem Cell Res. Ther..

[19] Peyrecave-Capo X., Saulnier N., Maddens S., Gremillet B., Desjardins I. (2022). Equine Umbilical Cord Serum Composition and Its Healing Effects in Equine Corneal Ulceration. Front. Vet. Sci..

[20] Gama K.B., Santos D.S., Evangelista A.F., Silva D.N., de Alcântara A.C., Dos Santos R.R., Soares M.B.P., Villarreal C.F. (2018). Conditioned Medium of Bone Marrow-Derived Mesenchymal Stromal Cells as a Therapeutic Approach to Neuropathic Pain: A Preclinical Evaluation. Stem Cells Int..

[21] Vizoso F.J., Eiro N., Cid S., Schneider J., Perez-Fernandez R. (2017). Mesenchymal Stem Cell Secretome: Toward Cell-Free Therapeutic Strategies in Regenerative Medicine. Int. J. Mol. Sci..

[22] An S., Anwar K., Ashraf M., Lee H., Jung R., Koganti R., Ghassemi M., Djalilian A.R. (2023). Wound-Healing Effects of Mesenchymal Stromal Cell Secretome in the Cornea and the Role of Exosomes. Pharmaceutics.

[23] Saccu G., Menchise V., Gai C., Bertolin M., Ferrari S., Giordano C., Manco M., Dastrù W., Tolosano E., Bussolati B. (2022). Bone Marrow Mesenchymal Stromal/Stem Cell-Derived Extracellular Vesicles Promote Corneal Wound Repair by Regulating Inflammation and Angiogenesis. Cells.

[24] Fernandes-Cunha G.M., Na K.S., Putra I., Lee H.J., Hull S., Cheng Y.C., Blanco I.J., Eslani M., Djalilian A.R., Myung D. (2019). Corneal Wound Healing Effects of Mesenchymal Stem Cell Secretome Delivered Within a Viscoelastic Gel Carrier. Stem Cells Transl. Med..

[25] El-Husseiny H.M., Mady E.A., Helal M.A.Y., Tanaka R. (2022). The Pivotal Role of Stem Cells in Veterinary Regenerative Medicine and Tissue Engineering. Vet. Sci..

[26] Mendt M., Rezvani K., Shpall E. (2019). Mesenchymal Stem Cell-Derived Exosomes for Clinical Use. Bone Marrow Transplant..

[27] Meissner J.M., Chmielińska A., Ofri R., Cisło-Sankowska A., Marycz K. (2024). Extracellular Vesicles Isolated from Equine Adipose-Derived Stromal Stem Cells (ASCs) Mitigate Tunicamycin-Induced ER Stress in Equine Corneal Stromal Stem Cells (CSSCs). Curr. Issues Mol. Biol..

[28] Harrell C.R., Fellabaum C., Jovicic N., Djonov V., Arsenijevic N., Volarevic V. (2019). Molecular Mechanisms Responsible for Therapeutic Potential of Mesenchymal Stem Cell-Derived Secretome. Cells.

[29] Freitas N.P.P., Silva B.D.P., Bezerra M.R.L., Pescini L.Y.G., Olinda R.G., Salgueiro C.C.d.M., Nunes J.F., Martins J.A.M., Neto S.G., Martins L.T. (2023). Freeze-Dried Platelet-Rich Plasma and Stem Cell-Conditioned Medium for Therapeutic Use in Horses. J. Equine Vet. Sci..

